# Endophytic microbes from Nigerian ethnomedicinal plants: a potential source for bioactive secondary metabolites—a review

**DOI:** 10.1186/s42269-021-00561-7

**Published:** 2021-06-08

**Authors:** Chijioke E. Ezeobiora, Nwamaka H. Igbokwe, Dina H. Amin, Udoma E. Mendie

**Affiliations:** 1 Department of Pharmaceutical Microbiology and Biotechnology, Faculty of Pharmacy, University of Lagos, Lagos, Nigeria; 2 Department of Microbiology, Faculty of Science, Ain Shams University, Cairo, Egypt

**Keywords:** Endophytes, Medicinal plants, Isolation, Bioactive metabolites, Biosynthetic gene clusters

## Abstract

**Background:**

Endophytes are highly beneficial species of microbes that live in symbiosis with plant tissues in the setting. Endophytes are difficult to isolate in their natural environment, and they are understudied despite being a rich source of bioactive molecules. There are varieties of new infectious diseases emerging across the world, necessitating a constant and expanded search for newer and more efficient bioactive molecules. Nigeria is known for its biodiversity in ethnomedicinal plants, yet these plants are understudied for endophytic microbes harbouring novel bioactive molecules.

**Main body:**

Endophytes are a source of novel organic natural molecules and are thought to be drug discovery frontiers. Endophyte research has contributed to the discovery of possible anticancer agents following the discovery of taxol. Endophyte research has contributed to the discovery of possible drug compounds with antimicrobial, antioxidant, antiviral, antidiabetic, anti-Alzheimers disease and immunosuppressive properties among others. These breakthroughs provide hope for combating incurable diseases, drug resistance, the emergence of new infectious diseases, and other human health issues. Finding new medicines that may be effective candidates for treating newly emerging diseases in humans has a lot of promise. Most studies have been on fungi endophytes, with just a few reports on bacterial endophytes. The biology of endophytic bacteria and fungi, as well as endophytic microbes isolated from Nigerian medicinal plants, their isolation methods, identification by morphological and molecular methods, fermentation, purification, identification of bioactive compounds and biosynthetic gene clusters are all covered in this study.

**Conclusion:**

In Nigeria, the sourcing and isolation of endophytes harboring biosynthetic gene clusters are still understudied, necessitating a rigorous quest for bioactive molecules in endophytes inhabiting various ethnomedicinal plants.

## Background

Nigeria is rich in plant biodiversity, and these plants are home to millions of endophytic microbial species, which give the ability to discover a wide range of biologically significant compounds while also providing a sustainable source of natural products (Eze et al. 2018; Okoye et al. 2015; Abba et al. 2016). According to the World Health Organization (WHO), infectious diseases continue to emerge and spread, and a lack of new antimicrobials jeopardizes global efforts to combat new and drug-resistant infections (2020). Hundreds of millions of recorded cases of covid-19, a recent pandemic that has afflicted the world and claimed the lives of 2.7 million people as recorded by the WHO (2021). This necessitates a massive quest for newer bioactive and antimicrobial compounds. Endophytic fungal populations of Nigerian medicinal plants have been studied and their enormous potential as sources of novel bioactive molecules has been highlighted, as well as the need to further explore Nigeria's plant biodiversity for endophytes producing biologically significant molecules (Ebada et al. 2016; Okezie et al. 2017; Akpotu et al. 2017). Plant-associated microorganisms called endophytes live in symbiosis with their host plants' living tissues. Many microorganisms, such as fungi, bacteria, and actinomycetes have been discovered in endophytic relationships with plants. Biosynthetic gene clusters (BGCs) have been found in these species. Endophytic fungi and bacteria live in plant tissue without causing disease symptoms, but they provide effective protection against phytopathogens (Gond et al. 2014). This allows them to generate a wide range of secondary metabolites, many of which have important biological activities and can be studied further for human health benefits. Secondary metabolites produced by endophytic association may be used to develop new compounds to combat the threats posed by multidrug-resistant (MDR) and prodrug-resistant (PDR) microbial strains, as well as newer emerging disease conditions. Antimicrobial resistance is steadily increasing, prompting many antimicrobial experts to expect a return to the pre-antibiotic era (Blaskovich and Coope 2014, 2017). Endophytic fungi and actinobacteria have been established as sources of bioactive secondary metabolites that could be used to fight resistant microorganisms (El-sayed et al. 2019). Highly efficient, less harmful, and environmentally friendly chemicals must be produced, discovered, and investigated from natural or synthetic sources (Tenguria et al. 2018). Fungal endophytes are the most common form of plant endophyte, and they generate an enormous amount of secondary metabolites (Arnold and Engelbrecht 2007; Joseph and Mini 2011). Endophytes contain several chemical compounds, including saponin, alkaloids, benzophenones, phenolic acids, and others (Cragg and Newman 2013). Endophytes contain secondary compounds that are antiviral, anticancer, antidiabetic, and immunosuppressive in nature (Ali and Rante 2018). Seasonal, climatic, and political factors have caused some setbacks in traditional methods for extracting bioactive compounds from natural sources over the years. The environmental issues that researchers face during extraction necessitate novel approaches to obtaining these compounds. As a result, improved methods involving a variety of endophytic species can be cost-effective and provide a viable option for discovering a large and sustainable source of bioactive compounds (Madhusudhan et al. 2015). The large-scale processing of taxol (a valuable anticancer drug) from the bark of the yew plant used microbial fermentation. It addressed the issue of the yew plant's slow growth and endangered status (Zhou et al. 2010).

Endophytes of medicinal plants have recently gotten a lot of attention because they appear to contain natural products that are beneficial to humans (Gunatilaka 2006). Several of these medicinal plants have been tested for endophytes all over the world (Li et al. 2008). Nigeria is not exempt in this respect. Several researchers in Nigeria have worked with a variety of native medicinal plants in pursuit of endophytes that could develop antimicrobial, anti-inflammatory, anticancer, antioxidative, and anti-leishmanicidal bioactive compounds.

The whole world is currently struggling with a variety of emerging infectious diseases. The latest covid-19 pandemic necessitates detailed research into effective antimicrobials. Several researchers are studying various infectious diseases to better understand and treat them using various natural and chemical formulations (Mane et al. 2017). Endophytes are one of those natural sources of bioactive agents. Researchers are attempting to isolate new bioactive compounds from newer species of endophytes for medicinal, agricultural, and industrial uses, despite their failure to clarify their origins, pathways, and mechanisms of action (Garg et al. 2011). It is therefore critical to encourage researchers to isolate novel bioactive compounds (Zuccaro et al. 2011).

Isolation, fermentation, extraction and structural elucidation are all steps in the process of discovering new bioactive compounds from endophytes. Scientists are interested in endophytic bioactive research because of the ecological, structural diversity and complexity of isolated compounds (Yuan et al. 2011). Crude natural products are mainly used in the pharmaceutical industry and are used to make semi-synthetic natural products (Suryanarayanan 2011; Mane et al. 2018). Pharmaceutical biotechnology includes the bioprospecting of fungal and bacterial endophytes for useful compounds. As a result, this analysis was performed to provide researchers with the knowledge they need to perform endophyte research.

## Main text

### Biosynthetic potentials of endophytes

Endophytes are currently used to make essential medicinal, chemical, and agricultural compounds that support humans. Endophytes contain high-value medicinal compounds such as taxol, podophyllotoxin, camptothecin, vinblastine, and vincristine, among others. As opposed to the traditional approach of using plants, which is non-abundant and uneconomical, endophytes as sources of bioactive compounds are less costly (Kumaran et al. 2010; Kumar et al. 2013). Endophyte-associated plants are known to develop metabolites that cause pathogen resistance. Also, secondary metabolites formed by these endophytes, such as antibiotics and lytic enzymes, provide defence against a variety of pathogens. Endophytes play an important role in pest and insect control in agriculture (Ahmed et al. 2012; Santos et al. 2003; Arnold et al. 2003). As compared to nonsymbiotic plants, plants associated with endophytes have been found to easily adapt and withstand abiotic stresses such as drought, salt, and heat (Gao et al. 2010; Jalgaonwala et al. 2011).

Endophytes are noted for developing a wide variety of biologically active natural products, which are classified as lignans, hormones, alkaloids, isocoumarins, terpenoids, phenylpropanoids, quinones, lactones, phenol and phenolic acids, aliphatic metabolites and so on. The study of secondary metabolites produced by endophytes of various host plants has become increasingly common around the world. Endophytes have developed several natural compounds with interesting biological properties such as antibiotics, antiviral, anticancer, antioxidants, insecticidal, antidiabetic, immunosuppressive properties and so on (Clay and Schardl 2011). It was also proposed that endophyte-derived antibiotic biomolecules could be used as a replacement for several chemically produced antibiotics which have recently been plagued by numerous drawbacks due to an increase in antimicrobial resistance (Waller et al. 2005; Madhusudhan et al. 2015; Padhi et al. 2013; Yu et al. 2010; Stinson et al. 2003; Strobel 2003). In Nigeria, there are few studies on the isolation of various bioactive compounds from endophytes. Table 1 shows few recent studies on isolated endophytes in Nigeria and their bioactive compounds.

**Table 1:** Reports of isolated endophytes from Nigerian plants

Compounds	Plant name	Plant part	Endophyte	Growth media	Method of urification	Activity	References
Acropyrone, Indole-3-acetic acid	*Chromolaena. odorata*	Leaves	Endophytic fungi	PDA	HPLC	Antimicrobial, Antioxidant	Buzugbe et al. (2018)
Glucobrassin, Ferulic acid, 4-Methoxybenzaldehyde, 12 Hydroxy-16-scaleren, 12-o-Deacetyl-12-episcalarin, Ixoside, Citreodrimene F, Cytosporin D	*Azadirachta indica*	Leaves, Stem	*Aureobasidium* sp*., Sodaria* sp., *Aspergillus* sp., *Penicillium* sp.	Rice medium, MEA	HPLC–DAD	Antimicrobial	Ujam et al. (2011)
Citreoisocoumarin, Citreoisocoumarinol, Questinol, Hydroxyemodin, Acropyrone, Nigricinol, Cladosporin	*Catharanthus roseus*	Leaves, Stem	Endophytic fungi	Rice medium, PDA	HPLC–DAD analysis	Antimicrobial	Akpotu et al. (2020)
Protocathechuic acid, Indole-3-acetic acid, Acropyrone	*Citrus jambhiri*	Leaves	Endophytic fungi	Rice media, PDA	HPLC analysis	Antibacterial	Eze et al. (2018)
Citreoisocoumarinol, Acropyrone, Citreoisocoumarin	*Psidium guajava*	Leaves	*Nectria* sp., *Microdochium bolleyi, Fusarium tricinctum, Penicillum* sp.	MEA, Rice medium	HPLC analysis	Antioxidant, Anticancer	(2018)
Indole-3-acetic acid, Indole-3-carbeldehyde	*Carica papaya*	Leaves	Endophytic fungi	Rice medium, MEA	HPLC analysis	Antimicrobial	Abba and Nduka (2016)
Ethyl-4-hydroxyphenyl acetate, Ferulic acid, Ruspolinone	*Newbouldia laevis*	Leaves	Endophytic fungi	Rice medium, MEA	HPLC analysis	Antibacterial	Okezie et al. (2017)
Acropyrone	*Loranthus micranthus*	Leaves	*Aspergillus* sp.	PDA	HPLC analysis	Antibacterial, Antioxidant	Akpotu et al. (2017)
Protocatechuic acid, Cladosporin, Scytalone	*Ocimum gratissimum*	Leaves	Endophytic fungi	MEA	HPLC	Antimicrobial	Abba et al. (2018)
4-Hydroxy phenyl acetic acid, p-Methoxycoumaric acid, Indole-3-acetic acid, Acropyrone	*Vernonia amygdalina*	Leaves	Endophytic fungi	MEA	HPLC–DAD analysis	Antimicrobial	Abba et al. (2018)
Citreoisocumarinol, Cladosporin, Acropyrone	*Euphorbia hirta*	Leaves	Endophytic fungi	Rice medium, MEA	HPLC–DAD	Antimicrobial, Antioxidant	Abonyi and Eze (2018)
Phenolic compounds	*Annona muricata*	Leaves	*Pseudofusicoccum* sp.	Rice medium	HPLC	Antimicrobial	Nwachukwu et al. (2018)
p-Hydroxyphenyl acetic acid, Ferulic acid	*Moringa oleifera*	Leaves	Endophytic fungi	PDA, Rice media	HPLC analysis	Antimicrobial, Antioxidant, Anticancer	Garba et al. (2018)
Acropyrone, 4-Hydroxy phenylacetic acid, Indole-3-acetic acid	*Picralima nitida*	Leaves	*Curvularia* sp.	PDA, rice medium	HPLC	Antimicrobial	Morare et al. (2018)
Fatty acid ester, 6-(5-Ethoxypentyl-2-methylhex-2-enedioate	*Sclerocarya birreas*	Stem, Bark	*Fusarium oxysporum*	PDA	Silica gel column chromatography	Antimicrobial	Ujam et al. (2020)

### Endophytes as potential sources for curbing new emerging infectious diseases.

Infections that have recently emerged, especially viral infections like Severe Acute Respiratory Syndrome (SARs-CoV or SARs) and Middle East Respiratory Syndrome (MERS-CoV or MERS), have caused regional and global outbreaks (Xu et al. 2020). At the end of 2019, a novel coronavirus, previously known as SARS-CoV-2, was reported as the cause of pneumonia in Wuhan, a city in China's Hubei Province. It has since spread to China and the rest of the world and it is now classified as a global health emergency (Xu et al. 2020).

Antiviral metabolites discovered and generated by endophytes have emerged as an exciting area in viral therapeutic and antiviral drug growth. Vaccines against CoVs are being produced all the time to reduce the prevalence of virally transmitted diseases. Natural bioactive compounds with potential as protease inhibitors and immunomodulators are abundant in endophytic microbes (Okoye et al. 2015). They have shown a lot of promise as possible candidates for antiviral drugs or alternative and complementary medicals for the prevention and treatment of CoVs (Suwannarach et al. 2020; Naik 2018).

Paclitaxel, an anticancer agent derived from endophytic fungi of the genera Alternaria, Aspergillus, Beauveria, Cladosporium, Chaetomella, Fusarium, Guignardia, Monochaetia, Nodulisporium, and Pestalotia, has been studied to possess the potential to treat CoVs (Naik 2018; Ryang et al. 2019). Many endophytic bioactive compounds have been investigated for their anti-coronavirus properties (2021). Endophytes were used to isolate anti-CoVs compounds such as dimethyl asterriquinone D, ganolucidic acid A, ganoderic acid A, ganoderiol acid B, colossolactone A, E, G and V. These compounds have also been evaluated for anti-HIV and anti-cancer properties (Martinez-Montemayor et al. 2019; Dine et al. 2008).

### Culturing of endophytes

#### Selection of the plant material

The medicinal plants used in endophyte studies are chosen after a thorough literature review using the internet, books and journals. Most endophyte researchers source endophytic bioactive molecules from plants or parts of plants based on ethnomedicinal information or application (Tsuchida et al. 2002).

#### Collection of plant material

The medicinal plants chosen for endophytic research are often found in natural ecosystems and botanical gardens. Plant sections that are free of disease are often collected with a sterile scalpel and stored in zip-lock plastic bags or polythene bags until isolation is completed within 48 hours (Tsuchida et al. 2002).

#### Endophyte’s isolation, purification and subculturing

The first and most important step in isolating endophytes is surface sterilization. It is done to eliminate all epiphytes or surface microbes. Treatment of plant tissues with an oxidant or general sterilizing agent for a period, followed by 3–5 times sterile rinse is the most common process (Tsuchida et al. 2002). Surface sterilization of plant tissue, aseptic cutting of sterilized sections into segments, plating the sterilized segments onto isolation media, usually Potato Dextrose Agar (PDA), Malt Extract Agar (MEA) for fungi and Nutrient Agar (NA), Luria Bertani (LB) Agar for bacteria, are the most used isolation procedures. Actinomycetes, which are filamentous bacteria that can be isolated as endophytes, are also cultured on Starch Casein Agar (SCA) and Brain–Heart Infusion (BHI) agar enriched thioglycolate broth. Macerated plant tissues may also be streaked onto the required agar media. The sterilizing agent can theoretically destroy any microbe on the plant surface without affecting the host tissue or endophytic microorganisms. Since the conditions needed to destroy the last microbe on the plant surface may already be lethal to certain endophytic microorganisms, this is a difficult task. Furthermore, the sterilizing agent may penetrate plant tissue, killing endophytic microbes as a result. The following steps are involved in the isolation and purification of endophytic microbes from plant tissue:

1. Pre-treatment is the first step. It serves to remove adhering soil particles and most microbial surface epiphytes. It involves washing each plant's leaves, stems, and roots separately under the tap water (Tsuchida et al. 2002).
2. The second step involves the surface sterilization of the plant material. This entails soaking the freshly collected plant parts in slow running tap water for 15 min, then washing them in Tween 20 (1 drop in 200 mL sterile distilled water [SDW]) for another minute and then rinsing them three times in SDW in the laminar airflow cabinet. Sodium hypochlorite (1–5 percent for 2–10 min), ethanol (70–95 percent for 30 s to 4 min), hydrogen peroxide, and mercuric chloride (0.05–0.2 percent for 2–5 min) are all commonly used sterilizing agents. Surface sterilization with 2 percent sodium hypochlorite, 70% ethanol, and 0.1 percent mercuric chloride is commonly used in most published research (Tsuchida et al. 2002).

Plant material will be cleaned and cut into small segments after being washed several times under running tap water. Following surface sterilization, the plant content is rinsed with 70 percent ethanol (C_2_H_5_OH) for 30 s, 0.01 percent mercuric chloride (HgCl_2_) for 5 min, 0.5 percent sodium hypochlorite (NaOCl) for 2–3 min and finally sterile distilled water for 2–3 times. Before being placed in the isolation media, the plant material will be dried between the folds of sterile filter papers (Wright et al. 2012).

### Isolation of the endophytes

After proper drying, endophytes are isolated from surface-sterilized plant materials. They are normally cut into smaller pieces to reveal the tissue before placing them aseptically onto the isolation media. Fungi are usually isolated using potato dextrose agar medium supplemented with chloramphenicol (100 µg/ml), while endophytic bacteria are isolated using nutrient agar or Luria Bertani agar supplemented with fluconazole (30–50 µg/ml) (Tsuchida et al. 2002; Wright et al. 2012). Plant materials such as stems, fruits, and roots are usually cut vertically into small segments to reveal the inner surface before being inoculated on the required media plates (Tsuchida et al. 2002; Wright et al. 2012). All the plates used to recover fungi endophytes are held at 28 °C for two to three weeks and are checked for microbial growth regularly. The bacterial recovery petri plates (with inoculated tissue samples) are incubated at 37 °C ± 3 °C in the dark. Sub-culturing is achieved if microbial growth is detected. All endophytic cultures are purified before being moved to a freshly prepared media plate. Until incubation, parafilm tape is used to cover plates with plant tissues. For bacteria, the observations are normally made over 48 h while for fungi, they can take up to two weeks. Appropriate monitors in which no plant tissue is inoculated are also set up. The purified endophytic isolates are maintained by transferring onto appropriate agar slants at 4ºC till further use (Tsuchida et al. 2002).

#### Surface sterilization effectiveness test

There are three approaches for assessing the efficacy of plant surface-sterilization. They are as follows:

1. Using surface-sterilized plant tissue to imprint onto nutrient media.
2. Dipping the treated explants into the nutrient broth.
3. Culturing aliquots of water from the last rinsing onto nutrient media.

After incubation, no microbial growth should be visible on the medium if the test was carried out properly. Surface sterilization is considered complete in this situation and isolated microbes from the plant parts thereafter are known as endophytes. The isolated microorganisms can only be classified as endophytes after full surface sterilization of the plant tissue is verified.

#### Endophytes’ isolation media

The type of growth medium used has a direct effect on the amount and type of endophytic microorganisms isolated. Endophytic bacteria and fungi are isolated using nutrient agar (NA) and potato dextrose media respectively. Since nutrient agar contains no component that inhibits the growth of endophytic fungi, the media used to isolate endophytic bacteria are supplemented with an antifungal agent, such as nystatin or fluconazole, at a concentration of 30–50 μg/mL of each (Tsuchida et al. 2002). Endophytic fungi are isolated using potato dextrose agar (PDA) supplemented with 100 µg/ml chloramphenicol. It is fortified with chloramphenicol to suppress the growth of bacteria.

### Endophyte characterization

#### Endophyte morphological characterization (preliminary)

All the bacteria isolates' phenotypic characteristics, such as microscopic features, gram reaction, endospore staining, motility, catalase and oxidase activity are determined using standard procedures. Culture features, spore formations and mycelium are used to classify endophytic fungi. Tease mount slides using Lactophenol Cotton Blue reagent are usually prepared and observed at 40X and 100X magnifications (Wright et al. 2012).

#### Endophyte molecular identification

i. Genomic DNA extraction

Each endophytic isolate's genomic DNA is extracted from pure colonies using a fungal/bacterial DNA extraction kit that is controlled according to the manufacturer's instructions (Wright et al. 2012). The internal transcribed spacer (ITS) region of the extracted DNA is then amplified using polymerase chain reaction (PCR) using primers with specific base sequences under the following conditions: Initial denaturation at 90 °C for 15 min; denaturation at 95 °C for 1 min; annealing at 56 °C for 1 minute (Hu et al. 2012).

ii. Polymerase chain reaction (PCR) of the rRNA genes and sequencing experiments.

Polymerase chain reaction (PCR) and subsequent DNA sequencing are used in most new endophyte identification methods. Amplification of the Internal Transcribe Spacer (ITS) region, which is a repeating unit of DNA that encodes ribosomal RNA, is the best way to identify fungal endophytes while 16S rRNA sequencing is used for bacteria identification. If the amplified DNA is sequenced and compared to a database, it can be used to decide if the specimen is novel; if the sequence is recognized, the species can be identified (Tidke et al. 2017). The primers (16S-27F: 5'-AGAGTTTGATCMTGGCTCAG-3' and 16S-1492R: 5'- CGGTTACCTTGTTACGACTT-3') can be used to amplify the 16S rRNA gene of each bacteria endophyte. The internal transcribed spacer (ITS) region of the extracted DNA is also amplified using the primers ITS1 (with base sequences 5'-TCCGTAGGTGAACCTGCGG-3') and ITS4 (with base sequences 5'TCCTCCGCTTATTGATATGC-3') under the required conditions on the extracted fungi DNA. The base sequences of the PCR products are compared to the data in Gene bank (Hu et al. 2012; Amin et al. 2020).

#### Phylogenetic analysis

The gene sequences (base pairs) are screened for chimeras using DECIPHER, then Basic Local Alignment Search Tool (BLAST) is used to find closely related endophytic bacterial or fungal species (Amin et al. 2020), typically at the National Center for Biotech Information (NCBI). Using Multiple Sequence Comparison by Log Expectation (MUSCLE), highly related sequences with 96 to 100 percent identity are compared with endophyte sequences isolated. The Neighbour-Joining approach is often used to draw phylogenetic trees. Molecular Evolutionary Genetics Analysis (MEGA) is used to conduct evolutionary analysis. All endophyte sequences are deposited in GenBank with accession numbers (Amin et al. 2019).

### Secondary metabolites’ production by endophytes

Endophytes from bacteria and fungi are cultured separately in 1L Erlenmeyer flasks containing 500 mL of sterile nutrient broth media and 500 mL of potato dextrose broth media for metabolite development. The media should be shaken for 7 days at 200 rpm and 30 °C. Most isolated endophytic fungi are fermented in a 1L Erlenmeyer flask containing sterilized rice medium (prepared by autoclaving a mixture of 100 g rice and 100 ml distilled water) (Hu et al. 2012). The flasks are inoculated with pure culture agar blocks 3 mm in diameter and incubated for 21 days at 25–27 °C. The secondary metabolites are extracted with appropriate solvent e.g., ethyl acetate after the fermentation is finished and then concentrated under vacuum at 40 °C. The crude extract is obtained by drying the solvent-soluble fractions using a rotary evaporator (Hu et al. 2012). Endophytic fungi and bacteria crude extracts are now used to monitor for bioactivity.

### Purification of bioactive compounds extracted from endophytes

In endophytic biomolecule research, this is a critical step. After the endophytes have been successfully cultured, the fractions extracted from the growth media will be subjected to bioassays to validate the endophyte's suitability for isolating the required active compounds.

The most commonly used approach is liquid–liquid extraction, which involves injecting an organic solvent into the liquid media of an endophyte culture (Hu et al. 2012). For extraction, several solvents may be used alone or in combination, depending on the solubility of the desired metabolite. The most used solvents for extracting metabolites from culture broth are ethyl acetate, methanol, dichloromethane, acetone, hexane, and ethanol (Hu et al. 2012) which are then dried by flash evaporation. If further purification is needed, the obtained fractions will be subjected to chromatographic techniques such as TLC and HPLC. Finally, gas chromatography (GC), nuclear magnetic resonance (NMR) or mass spectrometry (MS) can be used to validate the recovered fraction.

The two most popular techniques for determining structure are NMR and MS. X-ray diffraction (XRD) is also being considered as a method for crystallizing biomolecule (Hu et al. 2012).

### Screening for natural product biosynthetic gene clusters

When looking for biosynthetic gene clusters, the polymerase chain reaction (PCR) approach is commonly used. Polyketide synthase I (PKS-1), polyketide synthase II (PKS-II), and non-ribosomal peptide synthetase (NRPS) are the three main target genes (Hu et al. 2012; Tidke et al. 2017; Amin et al. 2019). For PCR amplification, three sets of degenerate primers targeting biosynthetic genes are usually used: KSF (50-GTSCCSGTSSCRTGSSHYTCSA-30) and KSR (50-CGCTCCATGGAYCCSCARCA-30), which target polyketide synthase (PKS)-I KS and methyl malonyl transferase domains; KSaF (50-TSGCSTGCTTGGAYGCSATC-30) and KSaR (50-TGGAANCCGCCGAABCCGCT-30), targeting PKS-II KSa genes and A3F (50-GCSTACSYSATSTACACSTCSGG-30) and A7R (50-SASGTCVCCSGTSCGGTAS-30), targeting non-ribosomal peptide synthetase (NRPS) genes. The amplified PCR products are analyzed by electrophoresis on 1% agarose gels. A negative control without a DNA template is often included with each PCR (Kun et al. 2010).

### Future perspectives

The ability to use endophytes to produce useful biomolecules as an effective and long-term alternative to traditional methods of searching for these biomolecules opens the possibility of using them as an effective and long-term alternative to traditional methods of searching for these biomolecules. Industrial development of these valuable compounds through endophytes, on the other hand, is still a long way off. The quest for bioactive molecules in endophytes isolated from Nigerian ethnomedicinal plants have not been thoroughly investigated. Furthermore, the majority of previous research has focused on endophytic fungi. In the endophytic bacteria and actinomycetes’ study, there is still a large gap that needs to be filled. There is a decrease in biomolecule generation with successive subculturing under certain condition. This has greatly hampered industrial applications of endophytes. Because of the significant endophytic contact with the host plant, even slight changes in culture conditions can have a significant impact on secondary metabolite output. Identification of improved strains suitable for fermentation from natural sources, genetic modification of existing strains to boost productivity using biotechnological procedures and optimization of culture conditions for increased yields are all necessary. As a result, a thorough investigation is needed to comprehend the interactions that are suitable for the large-scale development of bioactive molecules through endophytes. Whole-genome sequencing studies of isolated endophytic microbes are also needed.

## Conclusion

Endophytes as a drug source can aid in the conservation of biodiversity and antimicrobial resistance since they are non-traditional drug sources. Endophytes may be able to replace conventional drug development approaches. Endophytes hold promise as a source of bioactive compounds with potential for human benefit. The sourcing and isolation of endophytes containing biosynthetic gene clusters are still understudied in Nigeria, so a thorough search for bioactive molecules from endophytes inhabiting various Nigerian ethnomedicinal plants is needed.

## Data Availability

Not applicable.

## Abbreviations

PDA: Potato dextrose agar
LB: Luria Bertani
HPLC: High performance liquid chromatography
HPLC-DAD: High performance liquid chromatography with diode array detection
MEA: Malt extract agar
BGCs: Biosynthetic gene clusters
CoVs: Coronaviruses
SARs: Severe acute respiratory syndrome
HIV: Human immunodeficiency virus
MERS: Middle East respiratory syndrome
SCA: Starch casein agar
BHI: Brain heart infusion
NA: Nutrient agar
MDR: Multidrug resistant
PDR: Prodrug resistant
DNA: Deoxyribonucleic acid
PCR: Polymerase chain reaction
ITS: Internal transcribed spacer
RNA: Ribonucleic acid
rRNA: Ribose ribonucleic acid
BLAST: Basic local alignment search tool
NCBI: National Center for Biotech Information
MUSCLE: Multiple sequence comparison by log expectation
MEGA: Molecular evolutionary genetic analysis
TLC: Thin layer chromatography
GC: Gas chromatography
NMR: Nuclear magnetic resonance
MS: Mass spectrometry
XRD: X-ray diffraction
PKS-I: Polyketide synthase I
PKS-II: Polyketide synthase II
NRPS: Non-ribosomal peptide synthetase
WHO: World Health Organisation
